# Early intervention with a small molecule inhibitor for tumor nefosis factor-α prevents cognitive deficits in a triple transgenic mouse model of Alzheimer’s disease

**DOI:** 10.1186/1742-2094-9-99

**Published:** 2012-05-25

**Authors:** S Prasad Gabbita, Minu K Srivastava, Pirooz Eslami, Ming F Johnson, Naomi K Kobritz, David Tweedie, Nigel H Greig, Frank P Zemlan, Sherven P Sharma, Marni E Harris-White

**Affiliations:** 1 P2D Bioscience, Cincinnati, OH, 45242, USA; 2 Veterans Administration-Greater Los Angeles Healthcare System and Department of Medicine, David Geffen School of Medicine at UCLA, 11301 Wilshire Boulevard, (151), Los Angeles, CA, 90073, USA; 3 National Institute on Aging, National Institutes of Health Laboratory of Neurosciences, Intramural Research Program, Baltimore, MD, 21224, USA

**Keywords:** Alzheimer’s disease, memory, neuroinflammation, thalidomide, thiothalidomide, tumor necrosis factor-alpha

## Abstract

**Background:**

Chronic neuroinflammation is an important component of Alzheimer’s disease and could contribute to neuronal dysfunction, injury and loss that lead to disease progression. Multiple clinical studies implicate tumor necrosis factor-α as an inflammatory mediator of neurodegeneration in patients with Alzheimer’s because of elevated levels of this cytokine in the cerebrospinal fluid, hippocampus and cortex. Current Alzheimer’s disease interventions are symptomatic treatments with limited efficacy that do not address etiology. Thus, a critical need exists for novel treatments directed towards modifying the pathophysiology and progression.

**Methods:**

To investigate the effect of early immune modulation on neuroinflammation and cognitive outcome, we treated triple transgenic Alzheimer’s disease mice (harboring PS1_M146V_, APP_Swe_, and tau_P301L_ transgenes) with the small molecule tumor necrosis factor-α inhibitors, 3,6′-dithiothalidomide and thalidomide, beginning at four months of age. At this young age, mice do not exhibit plaque or tau pathology but do show mild intraneuronal amyloid beta protein staining and a robust increase in tumor necrosis factor-α. After 10 weeks of treatment, cognitive performance was assessed using radial arm maze and neuroinflammation was assessed using biochemical, stereological and flow cytometric endpoints.

**Results:**

3,6′-dithiothalidomide reduced tumor necrosis factor-α mRNA and protein levels in the brain and improved working memory performance and the ratio of resting to reactive microglia in the hippocampus of triple transgenic mice.

In comparison to non-transgenic controls, triple transgenic Alzheimer’s disease mice had increased total numbers of infiltrating peripheral monomyelocytic/granulocytic leukocytes with enhanced intracytoplasmic tumor necrosis factor-α, which was reduced after treatment with 3,6′-dithiothalidomide.

**Conclusions:**

These results suggest that modulation of tumor necrosis factor-α with small molecule inhibitors is safe and effective with potential for the long-term prevention and treatment of Alzheimer’s disease.

## Introduction

Tumor necrosis factor-α (TNFα) is a pleiotropic cytokine originally recognized for its anti-tumor activity [1]. TNFα plays a pivotal role in a wide variety of events mediated by ligation and signaling through one of its cognate receptors, TNF-RI or TNF-RII. In the central nervous system (CNS), TNFα influences both developmental and pathophysiological processes. These include promoting apoptosis during neuronal target innervation [2], altering neurogenesis [3], influencing cell fate determination [4], neuronal differentiation [5] and neuronal morphology [6], augmenting synaptic transmission [7-11] and induction and perpetuation of the innate response during neurodegenerative conditions such as multiple sclerosis, Parkinson’s disease and Alzheimer’s disease (AD) (reviewed in [12]).

AD is a progressive neurodegenerative disease characterized by neurotoxic beta amyloid protein (Aβ), neuritic plaques, intraneuronal tau-containing neurofibrillary tangles, synaptic degeneration, neuronal loss, inflammation and diminished cognitive function. Within the AD neuritic plaque, Aβ peptides (Aβ_1-40_, Aβ_1-42_ and so on), derived from amyloid precursor protein (APP), induce inflammation and subsequent neuronal death [13,14]. Sustained, uncontrolled microglial cytokine production drives neuroinflammation-induced AD neurodegeneration [13,15-20]. Supporting this notion, multiple *in vitro* studies reveal that Aβ-stimulated microglia induce synaptic dysfunction and neuron death through an activated cytokine network [19,21]. Activated microglia produce several immune and inflammatory mediators (including TNFα, IL-1, IL-6) that activate membrane receptor-mediated intracellular processes in nearby neurons, causing dysfunctional nerve signaling and, ultimately, neuronal death [19,21]. Inflammatory mediators also activate nearby microglia, establishing a chronic self-propagating cycle of glial activation and neuronal death [15,16,22]. This self-propagating cycle may underlie the progressive accumulation of synaptic dysfunction and neurodegeneration that leads to the observed cognitive deficits in AD [13,15,16].

Clinical studies highlight the relevance of TNFα in AD. Zhao *et al.* examined TNFα cascade components in vulnerable neuroanatomic locations of postmortem AD brains, transitional cases diagnosed with mild cognitive impairment (MCI) and cognitively unimpaired, age-matched controls [23]. Cortical and hippocampal TNFα levels were significantly elevated in patients with MCI and with AD compared with age-matched controls. Cerebrospinal fluid (CSF) and serum studies suggest TNFα to be an early biomarker of MCI and AD progression [24,25]. TNFα levels in the CSF were 25-fold higher in patients with AD compared to age-matched controls [24-26] and MCI patients with high CSF TNFα levels progress rapidly to AD [27]. Elevated CSF TNFα levels correlate with clinical deterioration in patients with MCI and with AD [26], suggesting that a rise in CSF TNFα level precedes AD development.

Preclinical AD models demonstrate the deleterious role of TNFα in AD-associated pathogenesis and cognitive deficits. Mice receiving Aβ _1–40_ by intracerebroventricular injection show marked deficits in learning and memory concomitant with elevated hippocampal TNFα mRNA levels [28]. In several mouse models that recapitulate specific human AD-related pathologies, TNFα is upregulated, co-localized with amyloid plaques, and is neurotoxic. These include the Tg2576 [29], APPswe/PS1dE9 [30] and 3 × TgAD mouse models [31].

In the present study, we utilized the 3 × TgAD mouse model, which demonstrates age-dependent changes in entorhinal cortex TNFα mRNA levels that strongly correlate with learning and memory deficits. By 4 months of age, TNFα mRNA levels are elevated 5.3-fold [31] and at this age these mice display early memory retention impairments [32]. Importantly, this TNFα elevation is prior to the onset of overt extracellular amyloid or tau pathology in 3 × TgAD mice. At 6 months, entorhinal cortex TNFα mRNA levels are 14.8-fold greater and mice demonstrate significant deficits in spatial reference learning. Starting from 4 months of age, 3 × TgAD mice were treated with the small molecule TNFα inhibitor, 3,6′-dithiothalidomide (3,6′-DT), thalidomide (Thal) or vehicle until they were 6.5 months of age. The mice were subsequently tested for cognitive impairment using the eight-arm radial arm maze (RAM) and the brains analyzed by immunohistochemical, biochemical and flow cytometric techniques.

## Methods

### Cell culture: BV2 microglia and splenocytes

BV2 cells were maintained in culture medium (CM) consisting of Dulbecco’s modified Eagle medium (DMEM + L-Glutamine, ATCC Cat #30-2002; Manassas, VA, USA) with 10% FBS (ATCC Cat #30-2020), penicillin/streptomycin (10,000 IU to 10,000 μg/mL; ATCC Cat#30-2300) in a 5% CO_2_ incubator. Plated cells (30,000 cells/well; 96-well plate) were grown in CM. In all experiments, cells were treated with the indicated concentrations of 3,6′-DT, Thal or vehicle (dimethyl sulfoxide; DMSO) in the absence or presence of lipopolysaccharide (LPS; 1 ng/mL; serotype O55:B5 from *Escherichia coli*) in serum-free CM. The final concentration of DMSO was 1%. The media was collected at 24 h after drug and LPS stimulation, briefly centrifuged to remove floating cells and debris and stored at −20°C prior to ELISA analysis. Lactate dehydrogenase (LDH) assay (Promega CytoTox96 non-radioactive cytoxicity assay; Madison, WI, USA) was performed in triplicate on CM as per manufacturer’s protocol.

For analyses of spleen TNFα production, spleens from Non-Tg and 3 × Tg mice were mechanically dissociated on a wire mesh by crushing with a 10 mL syringe, the red blood cells depleted, and filtered through 70 μm nylon strainers (BD Biosciences, San Diego, CA, USA). Splenocytes (2.5 × 10^6^ cells/mL) were cultured in CM in triplicate for 24 h and TNFα secreted in the CM quantified by ELISA.

### Animal studies

C57/Bl6 male mice were used for LPS injection studies (same LPS serotype as used in the *in vitro* studies). In this study, homozygous 3 × TgAD mice expressing mutant human genes APPswe, PS1M146V and tauP301L (previously characterized by Oddo *et al.*[33]) and wild- type mice from the same hybrid background strain, 129/C57BL6, were used. Starting from 4 months of age, 3 × TgAD mice received a daily intraperitoneal (i.p.) injection of 50 mg/kg 3,6′-DT, Thal or vehicle (Solutol (Sigma-Aldrich, St. Louis, MO,USA) in saline). Mice were housed on a 12 h light and 12 h dark schedule. All mice were given access to food and water *ad libitum*. At 6 months of age, the cognitive ability of the mice was assessed. All procedures involving animals were approved by the Institutional Animal Care and Use Committee at the Veterans Administration-Greater Los Angeles Healthcare System.

### Radial arm maze

The RAM used in this study consists of eight equally spaced arms radiating from a small circular central platform. The arms were 35.0 cm in length, 5.0 cm in width and 9 cm high (ANY-maze; Stoelting Co., Wood Dale, IL, USA; Item 60150). The maze was elevated 94 cm above the floor with each arm and the central platform supported underneath by a small wood table. Extramaze cues that surrounded the maze included the experimenter, two stainless steel racks, one wall-mounted storage cabinet and a sink. The cues were kept in consistent positions throughout the experiment and the maze was uniformly lit from ceiling lighting.

### Behavioral procedure

After mice were food-deprived to 90% of their *ad libitum* body weight, behavioral training began. For the first phase of behavioral training, mice were habituated to the maze for seven consecutive days. During habituation, three sucrose pellets (10 mg; P. J. Noyes Company, Inc., Lancaster, NH, USA) were placed down each of the eight arms of the RAM. Mice were released to the center platform and allowed to explore all eight arms, and arm visits as well as sucrose pellet consumption were recorded. Mice remained on the maze for 5 min during each daily habituation trial. Mice that did not consume any sucrose pellets or freely explore the maze by the end of the habituation period were excluded from behavioral testing.

After habituation was complete, the second phase of behavioral training began. Four arms were randomly selected for each animal and baited at the far end of each arm. Mice were released from the center platform, and arm visits were recorded. The training trial was considered complete when all four pellets were consumed or 5 min had passed. Two types of errors were recorded; working memory errors were revisits to arms that had been previously baited on the same training trial, and reference memory errors were visits to any of the four arms that had never been baited. In this phase of training, mice were trained for 9 consecutive days. The maze was wiped clean after each training trial using paper towels that were dampened with water.

### Tissue collection

Twenty-four hours after the last 3,6′-DT, Thal or vehicle injection, animals were anesthetized with pentobarbital and cardiac perfused with HEPES buffer (10 mM HEPES, 137 mM sodium chloride, 4.6 mM potassium chloride, 1.1 mM potassium phosphate monobasic, 0.6 mM magnesium sulfate and 1.1 mM ethylenediaminetetraacetic acid) containing sodium vanadate (1 mM), sodium pyrophosphate (1 mM), sodium fluoride (50 mM), leupeptin (0.002 mM), aprotinin (0.154 µM), pepstatin (1.46 µM) and phenylmethylsulfonyl fluoride (287.36 µM). The hippocampus and cortex were dissected from one hemisphere and either snap-frozen in liquid nitrogen (half cortex) and stored in a −80°C freezer or stored at −20°C in RNA*later* (Ambion Inc., Austin, TX, USA) for PCR analysis. The contralateral hemisphere was immersion fixed in formalin (Fisher Scientific, Pittsburgh, PA, USA) for 24 h followed by paraffin embedding.

### Enzyme-linked immunosorbent assay

The levels of TNFα in culture media or mouse cortical or spleen supernatants were measured using a commercially available ELISA kit for mouse TNFα (BioLegend ELISA MAX; BioLegend, San Diego, CA, USA) according to manufacturer’s instructions. This kit detects optimally in the 10 to 1,000 pg/mL range. Standards ranged from 7.8 pg/mL to 500 pg/mL in all assays. Samples were appropriately diluted to fall within the standard range and not below.

In brief, 250 μL of tissue extraction reagent (Invitrogen, Camarillo, CA, USA; Cat # FNN0071), containing protease inhibitor cocktail (Sigma-Aldrich; Cat # P2714), was added to each tissue sample. Tissue was homogenized with 20 passes of a Teflon pestle homogenizer. Homogenates were centrifuged at 10,000 rpm for 10 min at 4°C and the resulting supernatants were removed and stored at −20°C until use.

### Real time quantitative PCR analysis of tumor necrosis factor-α gene expression

The samples were stored in RNA*later* (Ambion Inc.) at −20°C. Total RNA was extracted using TRI reagent (Sigma-Aldrich) and BCP (Molecular Research Center, Inc., Cincinnati, OH, USA) as a phase separation reagent. RNA was purified using Qiagen’s RNeasy Kit (Qiagen, Germantown, MD, USA) and was quantified spectrophotometrically. RNA (1 μg) was reverse transcribed to cDNA using RT^2^ First Strand Kit (Qiagen). Real time quantitative (q)PCR, using an ABI 7300 Sequence Detection System (Applied Biosystems, Foster City, CA, USA), was performed for quantification of low-density TNFα mRNA. The amounts of mouse TNFα mRNA were determined by amplification of the cDNA target using the RT^2^ qPCR Primer Assay for TNFα (Qiagen). To normalize the quantification of TNFα mRNA for possible differences in the amount of each cDNA template, 18 S rRNA served as a housekeeping gene. PCR amplifications of TNFα and 18 S rRNA genes were carried out in conjunction with RT^2^ qPCR SYBR Green Master Mix (Qiagen). Each cDNA sample was tested in triplicate. The following temperature parameters were cycled 40 times: 15 s at 95°C, 1 min at 60°C. Standard curves were constructed for 18 S rRNA as an internal standard and for the TNFα gene. The amounts of TNFα mRNA gene expression was normalized by division by the amount of 18 S rRNA mRNA in each sample and expressed as fold change in comparison to control values.

### Immunohistochemistry, thioflavin S and stereology

Formalin-fixed brains were embedded in paraffin and 35 μm thick coronal sections were mounted on glass slides. Immunohistochemical staining for anti-ionized calcium-binding adapter molecule 1 to identify microglia (Iba-1, 1:800; Wako Chemicals, Neuss, Germany) or monoclonal biotinylated 6E10 primary antibody (1:1000; Covance, San Diego, CA, USA) for total APP/Aβ was performed on these sections with the VectaStain Elite ABC kit (Vector Laboratories, Burlingame, CA, USA). Diaminobenzadine (Sigma-Aldrich) was used as the chromagen. For 6E10, antigen retrieval using 70% formic acid for 3 min was performed prior to antibody staining.

Design-based stereology was used to quantify Iba-1 and 6E10 positive cells in the hippocampus. We used the optical fractionator method, a stereological method of unbiased cell counting within a defined volume, using Microbrightfield (MBF) Bioscience’s Stereo Investigator software (Williston, VT, USA).. The optical fractionator technique estimates the number of cells by multiplying the sum of cells counted by the reciprocal of the fraction of the region sampled. Briefly, sections were viewed with a Nikon E600 microscope with a motorized stage, interfaced with a computer running Stereo Investigator 9.0. The total hippocampus (Iba-1) and CA1 to CA2 region (6E10) were identified on slide-mounted sections and delineated for each section of each animal under a 2× objective, based on the atlas of Franklin and Paxinos [34]. Tissue thickness was measured at each sampling site with a standard guard zone of 2 μm applied throughout. Every fifth section was analyzed for a total of eight sections per mouse, beginning at bregma −1.455 mm and ending at −2.680 mm for Iba-1, and from bregma −1.525 mm to −2.750 mm for 6E10. All sampling was done under a 60× oil immersion objective. In addition to 6E10+ cell counts, 6E10 staining density in the CA1 to CA2 region of the hippocampus was evaluated using Image Pro Plus 5.0.1.11(Bethesda, MD, USA).

For thioflavin S, three sections (every fifth section beginning at bregma −1.490 mm and ending at −1.840 mm) were incubated for 8 min in an aqueous solution of thioflavin S (1% w/v) and then differentiated twice with 80% ethanol for 3 min each, followed by another 3 min wash with 95% ethanol. Sections were rinsed three times with double distilled water and coverslipped with Vectashield fluorescence mounting medium (Vector Laboratories).

### Flow cytometry

Flow cytometry was performed on a single cell suspension of brain-infiltrating leukocytes that were isolated using a protocol based on the separation of microglia and leukocytes by density centrifugation, as previously described [35]. Briefly, following perfusion with ice- cold 1× Hank’s Balanced Salt Solution without phenol red to remove blood, the brain (without cerebellum) was homogenized in Roswell Park Memorial Institute medium using a dounce homogenizer with loose (pestle A) and tight-fitting (pestle B) pestles and the cell suspension layered over a discontinuous Percoll gradient. The cell layer at the 70% to 30% Percoll interface was transferred to a clean conical tube, diluted three times with 1 × Hank’s Balanced Salt Solutionand centrifuged to pellet the cells. The pellet was re-suspended in 1 mL of cell staining buffer in preparation for cell counting and flow cytometry.

The fluorescent-conjugated flow anti-mouse Abs allophycocyanin (APC)-conjugated anti-GR1 (RB6-8 C5) was from BioLegend. Peridinin-chlorophyll-protein-complex (PerCP)-conjugated anti-mouse CD45 (30-F11) was from BD Biosciences. APC-Cy7conjugated anti-mouse LY6G (1A8) and phycoerythrin (PE) -conjugated anti-mouse TNFα (MP6-XT22) were purchased from eBiosciences (San Diego, CA, USA). For flow cytometric evaluation, leukocytic cell populations (approximately 10^6^) were stained in a volume of 50 μL of 2% FBS/PBS containing a cocktail of three cell surface antibodies (anti-CD45, anti-Gr1 and anti-LY6G) for 30 min on ice and then stained for intracytoplasmic TNFα expression using BD Biosciences cytofix/cytoperm solution, according to the manufacturer’s instructions. The stained cell samples were acquired on a FACSScan or FACSCalibur flow cytometer (Becton Dickinson, San Jose, CA, USA) in the University of California, Los Angeles, Jonsson Cancer Center Flow Cytometry Core Facility. A total of 10,000 to 25,000 gated events were analyzed using FCS Express 3 (De Novo Software, Thornhill, ON, Canada). Cells stained with irrelevant isotype-matched antibodies and unstained cells served as controls. The cutoffs were set according to control staining.

### Statistical analysis

All data were normally distributed and are presented as mean values ± standard error of the mean. In the case of single mean comparisons, data were analyzed by unpaired Student’s *t* -test. In the case of multiple mean comparisons, the data were analyzed by one- or two-way analysis of variance (ANOVA) followed by Bonferroni multiple comparison tests using statistics software (Prism 5.0; GraphPad, LaJolla, CA, USA). *P-*values less than 0.05 were regarded to reflect a significant difference.

## Results

### Thalidomide and 3,6′-dithiothalidomide inhibit tumor necrosis factor-α production induced by lipopolysaccharide both in BV2 microglia cells *in vitro* and brain cortical tissue *in vivo*

**Figure 1:**
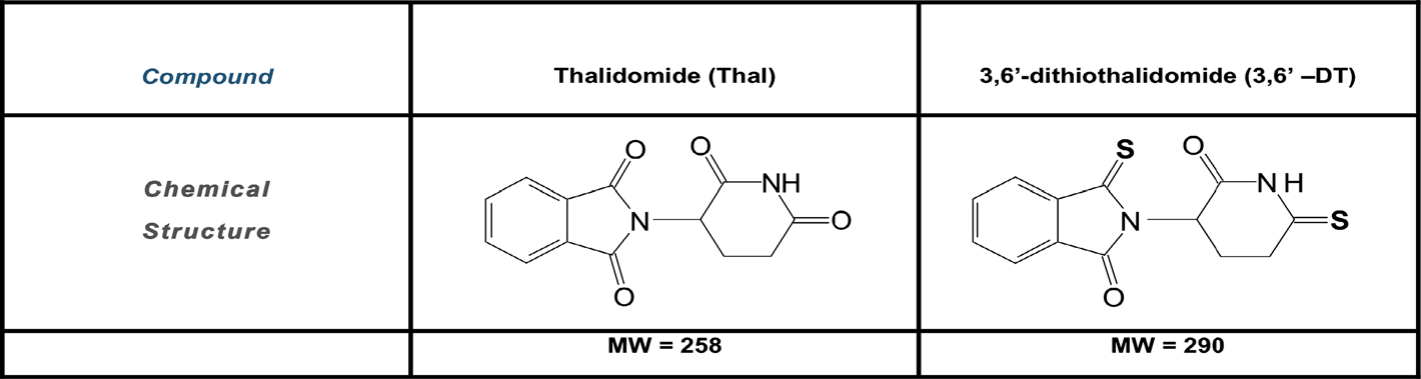
**The structures of thalidomide and 3,6**′**-dithiothalidomide.**

**Figure 2:**
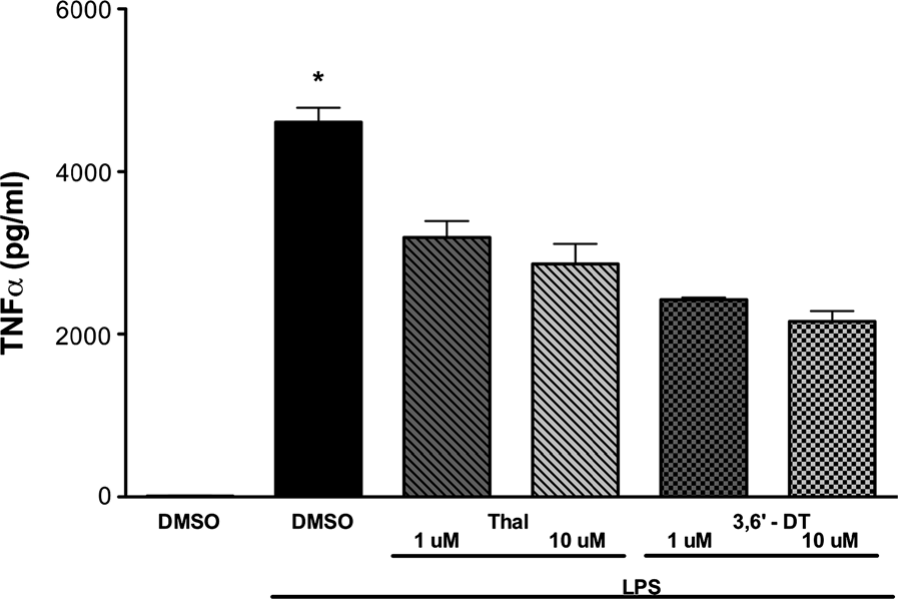
**Thalidomide and 3,6**′**-dithiothalidomide attenuate lipopolysaccharide-induced increase in tumor necrosis factor-α.** BV2 cells were treated with 1 ng/mL LPS ± Thal or 3,6′-DT for 24 h. Initial studies in BV2 cells demonstrate that both Thal and 3,6′-DT are effective at attenuating LPS-induced TNFα release into culture media. 3,6′-DT has an IC_50_ value of approximately 1 μM whereas the IC_50_ for Thal is > 10 μM. n = 6 per group. One-way analysis of variance: *P* < 0.0001; *P < 0.001 versus both drugs, both doses. LPS: lipopolysaccharide; Thal: thalidomide; TNFα: tumor necrosis factor-α; 3,6′-DT: 3,6′-dithiothalidomide.

Initial studies were conducted *in vitro* to verify the efficacy of Thal and 3,6′-DT (see Figure 1 for chemical structures) to inhibit TNFα. BV2 microglial cell cultures were treated with 1 ng/mL LPS with or without Thal or 3,6′-DT (1 and 10 μM). Culture media (n = 6 wells/treatment) was collected 24 h later and evaluated for TNFα protein levels via ELISA (Figure 2) and cytotoxicity by measuring LDH release into the media. One-way ANOVA revealed a significant effect of treatment (F_7__23_ = 51.03; *P* < 0.0001). Both Thal and 3,6′-DT significantly inhibited BV2 TNFα production at both concentrations compared with LPS alone (*P* < 0.0001 versus both drugs, both doses). 3,6′-DT was a more potent inhibitor, with a half maximal inhibitory concentration (IC_50_) value for TNFα inhibition of approximately 1 μM while the IC_50_ value of Thal was in excess of 10 μM, which is congruent with previous publications [36]. There was no increase in LDH in any treatment group including DMSO alone, LPS alone, Thal or 3,6′-DT alone or LPS plus Thal or 3,6′-DT (data not shown).

**Figure 3:**
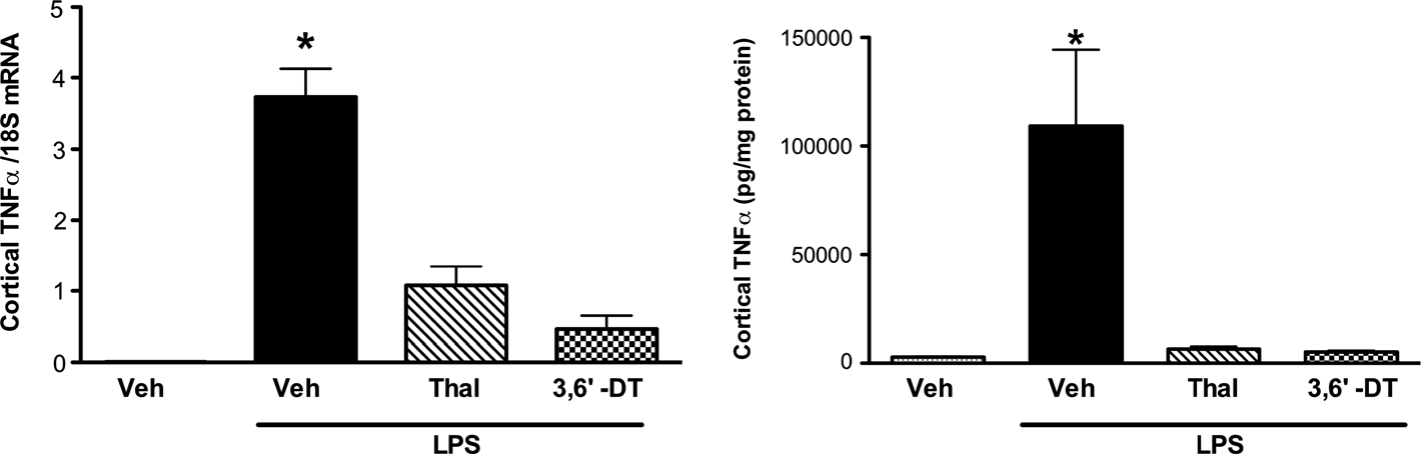
**3,6**′**-dithiothalidomide and thalidomide inhibited lipopolysaccharide-stimulated tumor necrosis factor-α gene and protein expression in C57 mice.** Mice were treated peripherally (intraperitoneal injection) with a single equivalent dose (100 mg/kg) of 3,6′-DT or Thal 30 min prior to a peripheral 5 mg/kg dose of LPS (intraperitoneal injection). Cortical tissue was harvested 4 hours after LPS injection. n = 6 per group. One-way analysis of variance: *P* < 0.001; *P < 0.001 versus all other groups. LPS: lipopolysaccharide; Thal: thalidomide; TNFα: tumor necrosis factor-α; 3,6′-DT: 3,6′-dithiothalidomide.

Both Thal and 3,6′-DT were effective at inhibiting brain cortical TNFα mRNA and protein levels in a systemic *in vivo* model of inflammation using LPS (Figure 3). C57 mice were given an i.p. injection of 100 mg/kg Thal or 3,6′-DT 30 minutes prior to an i.p. 5 mg/kg LPS injection. Four hours later, cortical tissue was harvested and analyzed by RT-PCR and ELISA. One-way ANOVA showed a significant effect of treatment on TNFα gene (F_4_,_29_ = 39.85; *P* < 0.0001) and protein (F_4_,_29_ = 34.58; *P* < 0.0001) expression. Both Thal and 3,6′-DT reduced LPS-induced brain cortical TNFα mRNA and protein levels to near vehicle-treated control values (*P* < 0.0001).

### 3,6′-dithiothalidomide, but not thalidomide, prevents cognitive impairment

**Figure 4:**
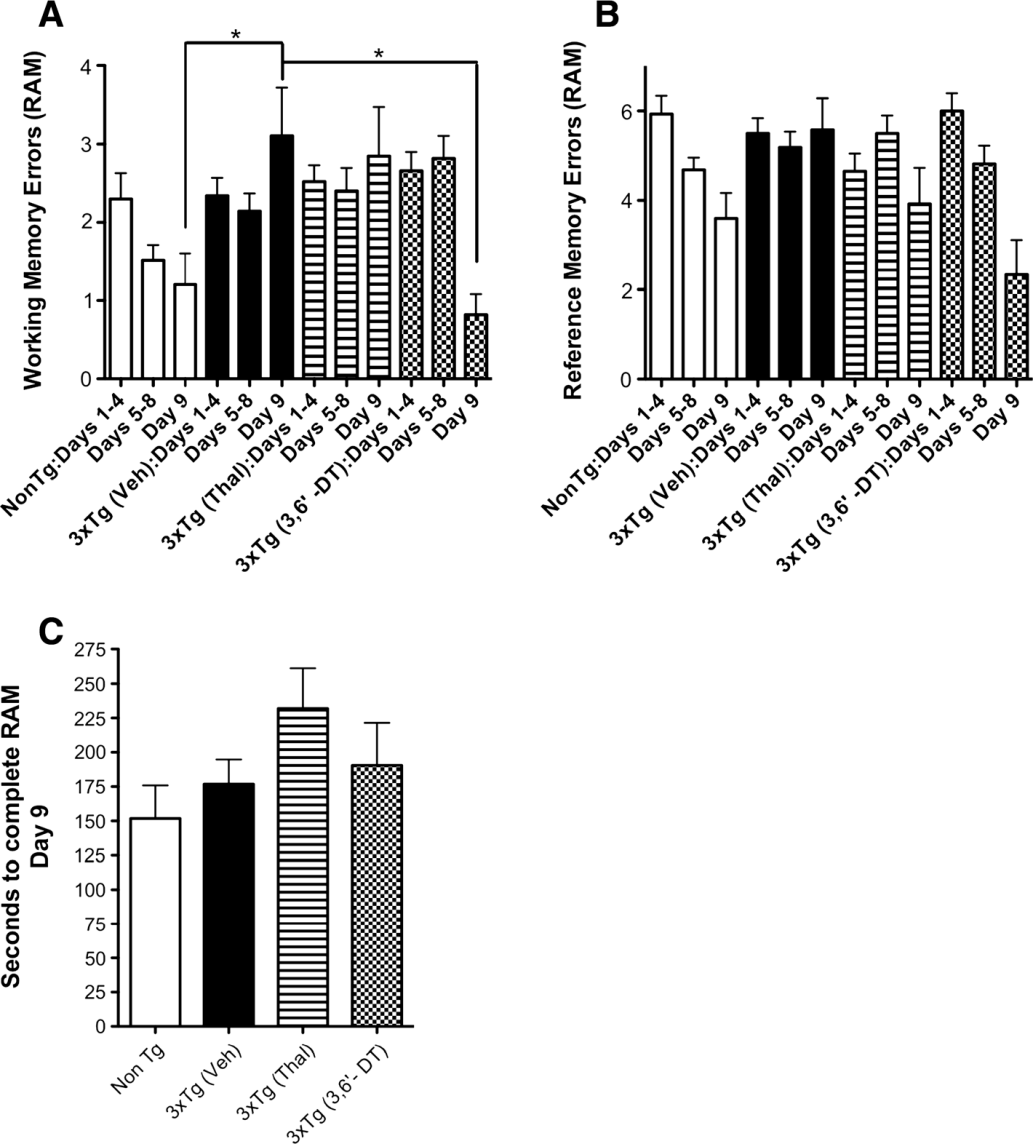
**3,6**′**-dithiothalidomide improves cognitive performance as assessed using the eight-arm radial arm maze in 3** × **Tg mice. (A)** 6.5-month-old 3 × Tg mice demonstrate a significant working memory deficit relative to age-matched Non-Tg mice (n = 15). Daily intraperitoneal administration of 50 mg/kg, 3,6′-DT for 2.5 months (4 to 6.5 months of age) significantly improved working memory errors (WME) in 3 × Tg mice (n = 11) compared with vehicle-treated (n = 14) or Thal-treated (50 mg/kg intraperitoneal injection; n = 11) 3 × Tg mice. No difference in WME was observed between 3, 6′-DT-treated 3 × Tg mice and age-matched Non-Tg mice. One-way analysis of variance: *P* = 0.0076. *P < 0.05. **(B)** Reference memory errors and **(C)** time to complete the radial arm maze on the last day of testing were not significantly different between groups. Thal: thalidomide; TNFα: tumor necrosis factor-α; 3,6′-DT: 3,6’-dithiothalidomide; WME: working memory errors.

Beginning at 4 month of age, 3 × Tg mice were treated with Thal, 3,6′-DT or vehicle for 2.5 months. There were no observable adverse effects of daily i.p. administration of Thal or 3,6′-DT. Mice were habituated to the RAM and were fully ambulatory and explored the RAM normally. Both working and reference memory errors were quantified during all acquisition sessions. Figure 4A,B represents the effect of treatment on working memory errors and reference memory errors made during the acquisition test, respectively. Repeated measures ANOVA showed a statistical effect of treatment on working memory errors (F_3_, _432_ = 2.92, *P* = 0.042, Figure 4A) and a significant interaction of ‘treatment by sessions’ (F_24_, _432_ = 2.38, *P* = 0.0003, Figure 4A). On day 9, 3 × Tg (vehicle) mice performed significantly worse than Non-Tg mice (*P < 0.05, Bonferroni post hoc analysis), and 3 × Tg (3,6′-DT) mice performed significantly better than 3 × Tg (vehicle) mice (*P < 0.05), indicating that spatial learning was impaired in vehicle-treated, but not impaired in 3,6′-DT-treated 3 × Tg mice. A similar statistical analysis revealed that reference memory errors decreased with time (F_8_, _432_ = 0.53, *P* = 0.6668, Figure 4B) but treatment did not have a significant effect (F_3_, _432_ = 260.4, *P* = 0.6488, Figure 4B). Figure 4 C indicates that there was no significant difference in time to complete the RAM on day 9 (F _3_,_47_ = 0.1635; *P* = 0.1635).

### 3,6′-dithiothalidomide treatment reduces brain and spleen tumor necrosis factor-α levels

**Figure 5:**
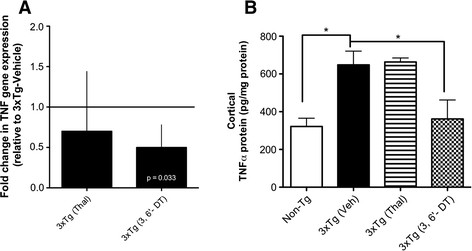
**Tumor necrosis factor-α gene and protein expression in the 3** × **Tg mouse. (A)** Fold-change (2^(− δ δCt)) is the normalized gene expression (2^(−δ Ct)) in the test sample (3 × Tg treated with Thal or 3,6′-DT) divided by the normalized gene expression (2^(−δ Ct)) in the control sample (vehicle-treated 3 × Tg). Fold-change values of less than one indicate a negative- or downregulation. Both Thal and 3,6′-DT downregulated TNFα gene expression but the value was significant only in the 3,6′-DT group (*P* = 0.033). **(B)** TNFα protein levels are doubled in the cortex of 3 × Tg mice compared with Non-Tg mice. 3,6′-DT, but not Thal, reduced TNFα protein levels near to Non-Tg levels in 3 × Tg mice. n = 5 to 8 per group. One-way analysis of variance (*P* = 0.062, *P < 0.05). Thal: thalidomide; TNFα: tumor necrosis factor-α; 3,6′-DT: 3,6’-dithiothalidomide.

**Figure 6:**
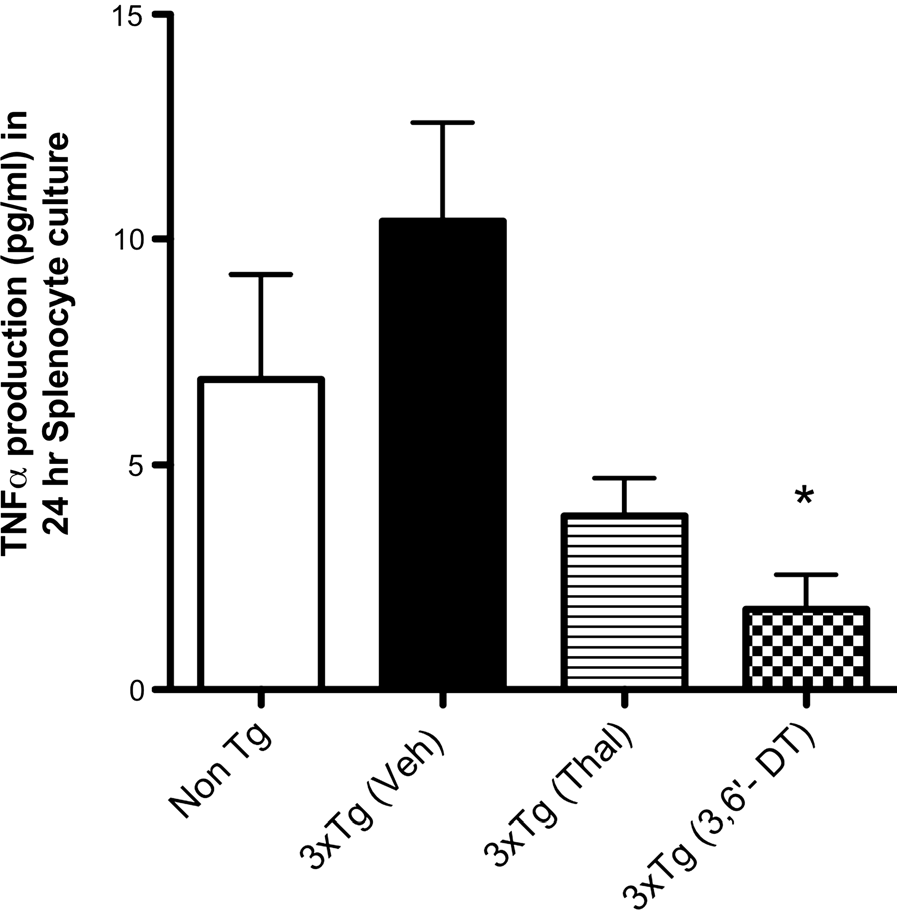
**Tumor necrosis factor-α protein from splenocytes isolated from Non-Tg and 3** × **Tg mice.** Splenocytes isolated from Non-Tg and 3 × Tg mice were cultured for 24 hours and TNFα levels measured by ELISA. 3,6′-DT-treated 3 × Tg mice had significantly reduced TNFα secretion compared with vehicle-treated 3 × Tg mice. One-way analysis of variance: *P* = 0.0184. *P < 0.05 versus 3 × Tg (vehicle). ELISA: enzyme-linked immunosorbent assay; 3,6′-DT: 3,6′-dithiothalidomide; Thal: thalidomide; TNFα: tumor necrosis factor-α.

A significant reduction in brain TNFα gene expression was observed in 3 × Tg mice treated with 3,6′-DT but not with Thal (Figure 5A; *P* = 0.033). There was a significant effect of treatment on TNFα protein in the cortex (ANOVA F_3_,_35_ = 4.956; *P* = 0.0062) with TNFα protein significantly decreased to near Non-Tg levels by 3,6′-DT (*P* < 0.05 3 × Tg (vehicle) versus 3 × Tg (3,6′-DT)) but not by Thal treatment (Figure 5B). In contrast, both Thal and 3,6′-DT were effective at reducing TNFα protein in the periphery as assessed by 24-h splenocyte production of TNFα (Figure 6). One-way ANOVA for treatment was significant (F3,13 = 5.374; *P* = 0.0184) with *P* <0.05 for 3 × Tg (vehicle) versus 3 × Tg (3,6′-DT). The reduction was not significant for 3 × Tg (vehicle) versus 3 × Tg (Thal).

### 3,6′-dithiothalidomide improves the ratio of resting to activated microglia

**Figure 7:**
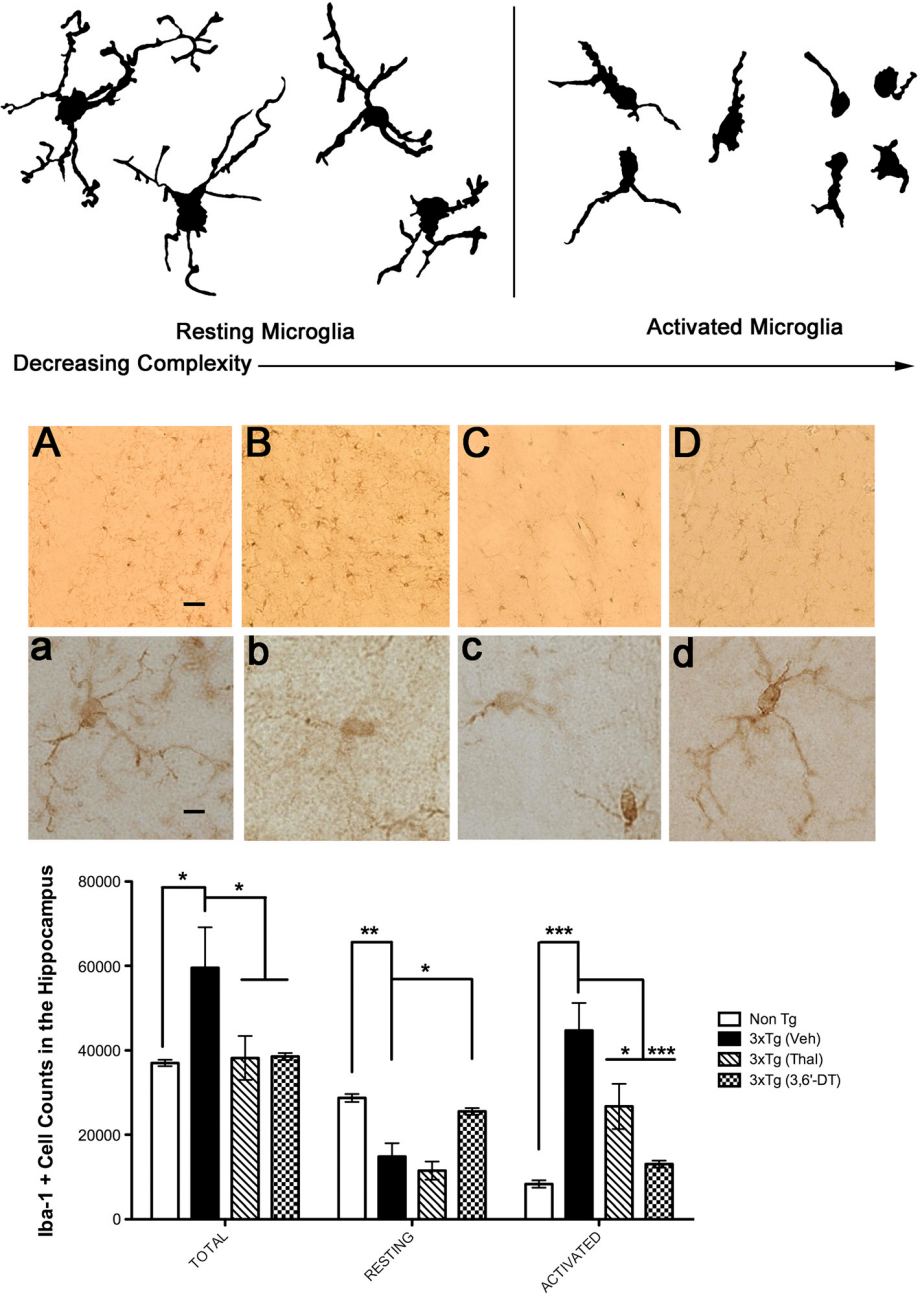
**Stereological analysis of ionized calcium-binding adapter molecule 1 staining in the hippocampus.** Unbiased stereological methods were used to analyze 35 μm sections, every fifth section through the hippocampus (total of eight sections per mouse; n = 3 to 4 per group). The top panel shows the morphological criteria used for designating resting versus activated Iba-1 positive microglia. Middle Panel: **(Aa)** Non-Tg; **(Bb)** 3 × Tg (vehicle); **(Cc)** 3 × Tg (Thal); **(Dd)** 3 × Tg (3,6′-DT) mice. Bottom panel: total, resting and activated cell counts. Bonferroni post hoc testing: *P < 0.05, **P < 0.01, ***P < 0.001. Bar equals 40 μm (A), 10 μm (a). 3,6′-DT: 3,6′-dithiothalidomide; Iba-1: ionized calcium-binding adapter molecule 1; Thal: thalidomide.

Using unbiased stereological methods, we examined changes in Iba-1 positive microglia in the hippocampus of 3 × Tg (vehicle, thalidomide- and 3,6′-DT-treated) and Non-Tg mice (n = 3 to 4 per group) and found a significant effect of treatment on total (F_3_,_8_ = 5.565; *P* < 0.0233), activated (F_3_,_8_ = 20.88; *P* = 0.0004) and resting (F_3_,_8_ = 17.17; *P* = 0.0008) microglia (Figure 7). Treatment of 3 × Tg mice with 3,6′-DT or Thal was effective at reducing the total number of Iba-1 positive brain microglia (Figure 7 Cc-Dd). Only 3,6′-DT increased the ratio of resting microglia to activated microglia (as assessed morphologically; see Figure 7, upper panel) resulting in a microglial morphological profile in the hippocampus that is more similar to the Non-Tg hippocampus.

### Amyloid precursor protein/amyloid beta peptide staining is not changed by treatment with thalidomide or 3,6′-dithiothalidomide

**Figure 8:**
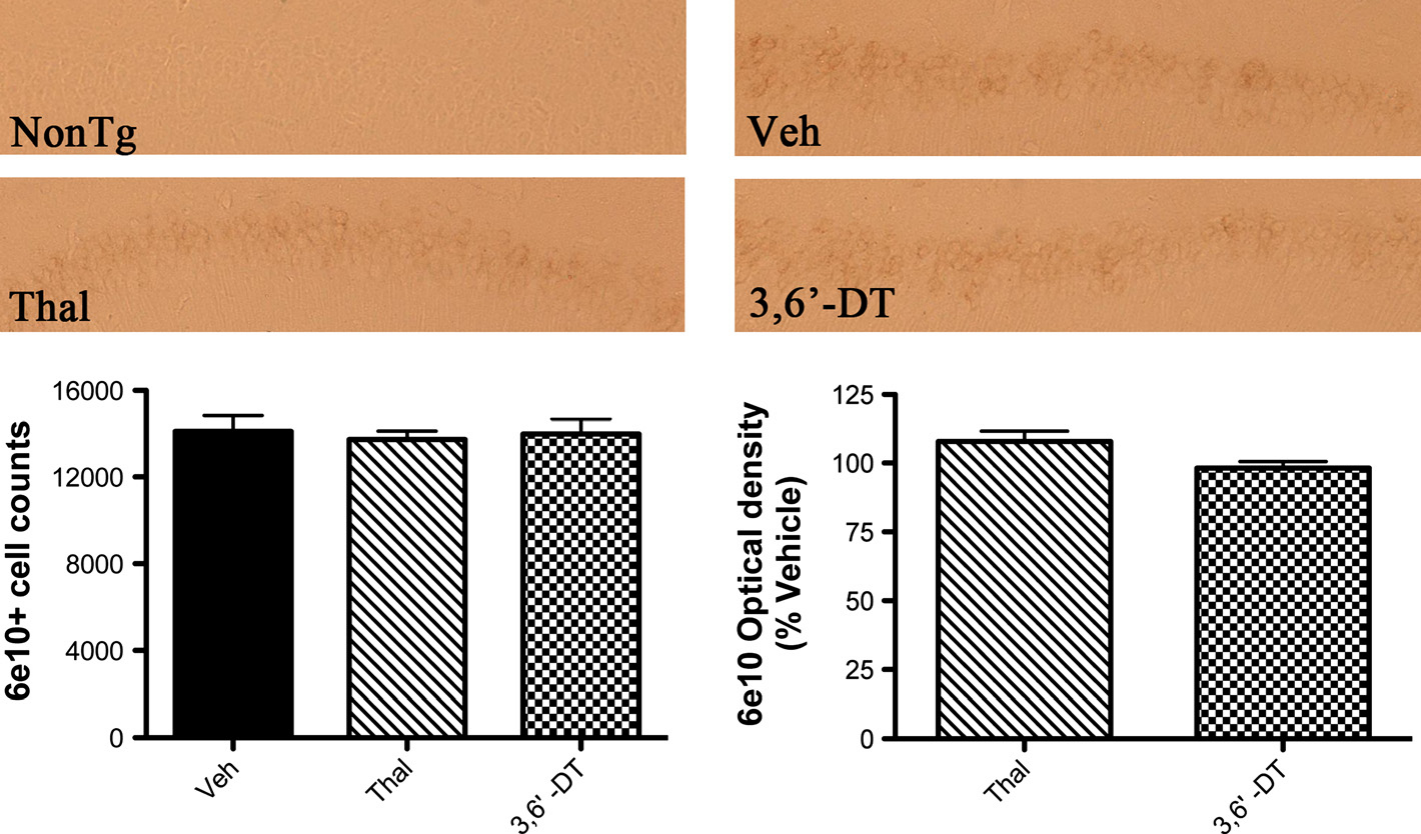
**Amyloid precursor protein/amyloid beta peptide staining is not changed in 3** × **Tg mice by 3,6**′**-dithiothalidomide or thalidomide treatment.** 6E10 immunohistochemistry was stereologically analyzed in the hippocampal CA1 to CA2 regions (left graph; n = 5 to 6 mice per treatment). There were no statistically significant differences in 6E10+ cell counts between treatment groups. Representative photomicrographs of each treatment group are shown. The same regions were also analyzed for optical density (right graph) and show that there were no differences between treatment groups.

The number of 6E10+ cells in the CA1 to CA2 region of the hippocampus was not changed by either Thal or 3,6′-DT treatment. Intraneuronal 6E10 staining was light at 6.5 months of age in the 3 × Tg mice with only an occasional diffuse plaque found and the majority of the staining was confined to cells in the hippocampus and cortex. Figure 8 shows representative sections of the CA1 to CA2 region of the hippocampus. Stereological counts of CA1 to CA2 did not reveal differences across treatment groups in either numbers of 6E10+ cells in this region or in 6E10 optical density. At 6.5 months of age, thioflavin S + deposits were not seen in the 3 × Tg mouse model [37] and none were observed in 6.5 month control 3 × Tg mice in this study. Treatment with Thal or 3,6′-DT did not alter this.

### 3,6′-dithiothalidomide reduces tumor necrosis factor-α in central nervous system-infiltrating leukocytes

**Figure 9:**
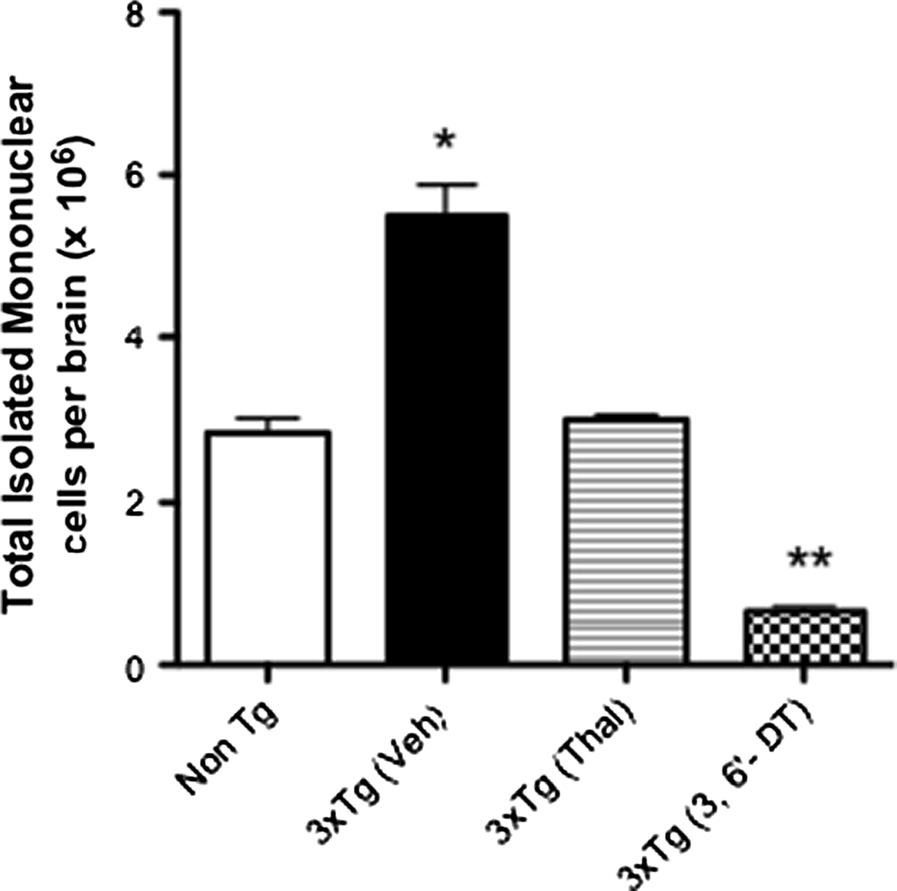
**3,6**′**-dithiothalidomide reduces central nervous system-infiltrating peripheral blood leukocytes.** Mononuclear cells were isolated from the whole brains of Non-Tg and 3 × Tg mice (vehicle-, Thal- and 3,6′-DT-treated; n = 3 to 4 per group) following 2.5 months of treatment and counted using a hemocytometer. One-way analysis of variance: *P* < 0.0001; *P < 0.001 versus Non-Tg, **P < 0.001 versus 3 × Tg (Thal). Thal: thalidomide; 3,6′-DT: 3,6’-dithiothalidomide.

Infiltrating leukocytes were isolated from whole brains to determine if treatment could alter the numbers of peripherally infiltrating cells (Figure 9; ANOVA F3,11 = 85.19; *P* < 0.0001). 3 × Tg mice have greater than twice the numbers of infiltrating leukocytes as Non-Tg mice (*P* < 0.001). These infiltrates were derived from whole brain homogenates and it is unknown to what extent these leukocytes migrated into the brain parenchyma or if they were maintained in the perivascular compartments of the brain. Both Thal and 3,6′-DT reduced the numbers of these cells (*P* < 0.0001 versus 3 × Tg (vehicle)). 3,6′-DT was more effective than Thal at reducing the numbers of infiltrating leukocytes (*P <* 0.001; 3 × Tg (3,6′-DT) versus 3 × Tg (Thal)), reducing the numbers well below Non-Tg cell numbers (*P* < 0.001; 3 × Tg (3,6′-DT) versus Non-Tg).

### 3,6′-dithiothalidomide decreased tumor necrosis factor-α in myelomonocytic/granulocytic cells

**Figure 10:**
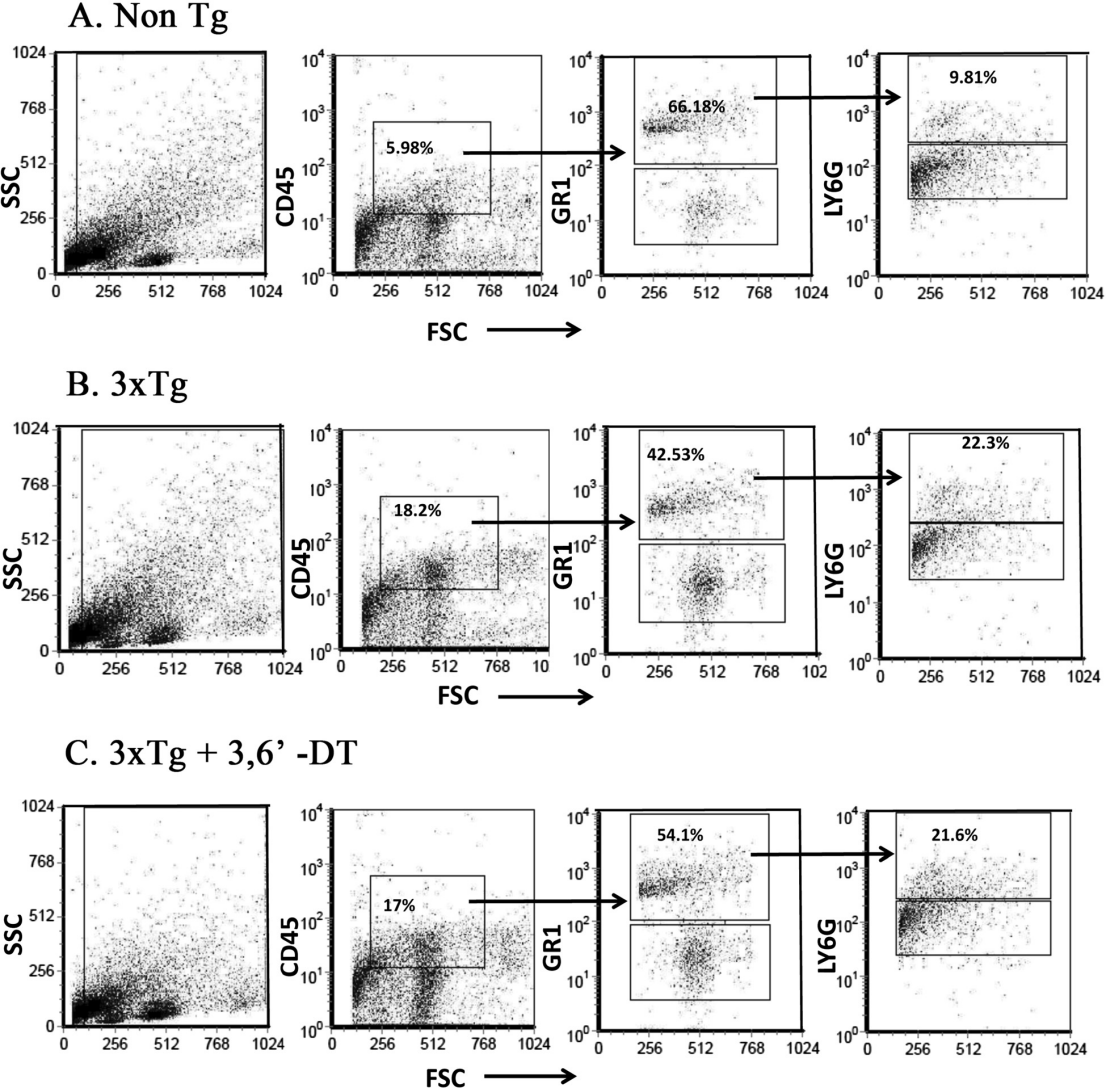
**3,6**′**-dithiothalidomide reduces tumor necrosis factor-α in central nervous system-infiltrating myelomonocytic/granulocytic leukocytes. (A)** Non-Tg mice; **(B)** 3 × Tg mice; **(C)** 3,6′-DT mice. CNS-infiltrating leukocytes from whole mouse brains (n = 3 to 4 per group) were isolated and evaluated for the presence of CD45_hi_ and myelomonocytes/granulocytes (CD45_hi_/Gr1^+^/Ly6G_hi_) by cell surface staining and flow cytometric analyses. There was a trend towards an increased percentage of CD45_hi_ cells and CD45_hi_/Gr1^+^/Ly6G_hi_ (not significant) in 3 × Tg mice (B) relative to Non-Tg (A) mice. 3,6′-DT (C) did not alter the percentages of these cell populations. TNFα expression in the total CD45_hi_ population and in the granulocyte population was increased in 3 × Tg mice relative to Non-Tg mice. 3,6′-DT treatment did not reduce TNFα expression in the total CD45 _hi_ population but specifically reduced TNFα expression in the CD45_hi_/Gr1^+^/Ly6G_hi_ population (*P* = 0.031). Flow cytometry results were quantified and are presented in Table 1.

CNS-infiltrating leukocytes were isolated and stained for fluorescence activated cell sorting (FACS) analysis to evaluate the changes in the CD45_hi_ population and their TNFα expression (Figure 10 and Table 1). There was a trend towards increased percentage of CD45_hi_ (ANOVA; *P* = 0.16) and CD45_hi_/Gr1^+^/Ly6G_hi_ (myelomonocytic/granulocytic; ANOVA *P* = 0.10) populations in the 3 × Tg relative to Non-Tg mice. TNFα production was increased in both the CD45_hi_ and the CD45_hi_/Gr1^+^/Ly6G_hi_ populations in the 3 × Tg mice relative to Non-Tg mice. 3,6′-DT reduced TNFα levels in the CD45_hi_/Gr1^+^/Ly6G_hi_ population (*P* = 0.0309).

**Table 1:** Central nervous system-infiltrating peripheral blood-derived leukocytes

	% CD45_hi_	%CD45_hi_/Gr1+/Ly6G_hi_	Intracellular TNFα^a^	
			CD45_hi_/Gr1+/Ly6G_hi_	
**Non-Tg**	6.7 ± 1.8	5.5 ± 2.0	-	-
**3** × **Tg**	14.3 ± 4.9	13.9 ± 4.4	493 ± 197.7	88.5 ± 16.7
**3** × **Tg (3,6′-DT)**	17.4 ± 1.9	13.6 ± 1.3	759 ± 75.3	28.3 ± 5.2
***P***	n.s.	n.s.	n.s.	0.0309

^a^TNFα is expressed as a percentage increase from Non-Tg values. *P*-value is 3 × Tg versus 3 × Tg (3,6′-dithiothalidomide). Results are expressed as the mean ± standard error; n = 3 to 4 mice per group. n.s.: not significant; TNFα: tumor necrosis factor-α.

## Discussion

There is a robust increase in TNFα expression levels in the CNS during numerous experimental models of both acute injury and chronic neurodegenerative disease, such as AD, suggesting a significant role for this cytokine in the injury or disease process [12,38]. Neuroinflammation begins early in AD and accompanies Aβ accumulation and neurodegeneration [39]. Still nebulous is whether this AD-related inflammatory response is advantageous or deleterious and what the best approach is to resolving the inflammatory tide while simultaneously allowing beneficial processes to continue. In the current study, we focus on the central role of TNFα and its modulation in inflammatory regulation and cognitive function in the 3 × Tg mouse model of AD.

Although there is ample evidence that TNFα plays a central role in brain development and homeostatic and repair mechanisms [40], many studies demonstrate a negative role for TNFα in AD pathology. APP/presenilin 1 (PS1) transgenic mice receiving short-term CNS infusion of anti-TNFα monoclonal antibody showed reduced tau pathology and amyloid plaque deposits [41]. Ligation of microglial CD40 with its cognate ligand, CD40 ligand (CD40L, expressed by activated astrocytes associated with beta-amyloid plaques [42]), synergistically activated microglia to produce TNFα in response to low levels of Aβ peptides. This form of microglial activation was deleterious, as it resulted in TNFα-dependent neuronal injury. Further, when mice deficient in CD40L were crossed with the Tg2576 mouse model of AD, abnormal phosphorylation of tau (an index of neuronal stress) was reduced prior to beta-amyloid deposition, suggesting that the CD40-CD40L interaction is an early event in AD pathogenesis [43]. However, complete abrogation of TNFα is not beneficial in the context of AD. Giuliani and coworkers used the PDAPP mouse model to demonstrate increased amyloid plaque burden and no cognitive improvement following chronic TNFα ablation [44]. The dual mission of TNFα may depend on the timing and progression of damage. In a model of traumatic brain injury, TNFα-null mice exhibited less severe cognitive and motor neuron impairments than wild type (WT) mice in the acute post-traumatic period [45]. While neurological functions recovered by 2 to 3 weeks post-injury in WT mice, TNFα-null animals still demonstrated motor deficits at 4 weeks and brain damage was significantly more extensive in TNFα-deficient mice. What remains unclear after these important studies is which approach to pursue in balancing the dual roles of the inflammatory response in AD. Our data indicate that long-term modulation with the small molecule TNFα inhibitor 3,6′-DT is safe, reduces CNS TNFα levels and improves cognitive function in the early stages of disease in the 3 × Tg mouse. It will be important to assess long-term dosing strategies that encompass later disease stages for safety and impact on the development of the classical neuropathological features of AD, such as tau pathology (not seen at the age of the mice in this study; see [33,37]) and amyloid accumulation. It is important to note that, at this early phase of the disease, treatment of 3 × Tg mice with either Thal or 3,6′-DT did not increase intraneuronal Aβ or Aβ plaque deposition.

TNFα has already been validated as a drug target with infliximab (Remicade), etanercept (Enbrel) and adalimumab (Humira) in clinical use. Short-term, extrathecal etanercept administration in patients with AD achieved significant cognitive and behavioral improvements [46-48]. As AD treatment necessitates chronic, long-term treatment, perispinal injections are neither practical nor safe in this context and the development of small, drug-like molecules to potently and safely inhibit TNFα is of significant clinical value. Thalidomide, a small molecule glutamic acid derivative demonstrating anti-TNFα actions, enhances the degradation of TNFα mRNA [49-51]. Recent preclinical studies indicate the therapeutic potential of employing thalidomide as an AD treatment. Daily treatment with thalidomide (20 mg/kg) improved recognition memory induced by Aβ(25–35) or Aβ(1–40) in mice [52]. These data suggest the practicability of small molecules that target TNFα as a therapeutic strategy against Aβ-mediated cognitive impairments. However, thalidomide’s use in humans is severely restricted by its well-documented side effects, such as somnolence, deep-vein thrombosis and peripheral neurotoxicity [53]. Thalidomide also has a high IC_50_ for TNFα inhibition, necessitating chronic high dosing to achieve significant clinical benefit while increasing risk for side effects in patients with AD.

In this report, a novel experimental TNFα synthesis inhibitor, 3,6′-DT [54,55], a lipophilic analog of the classic orally active thalidomide, was evaluated. 3,6′-DT was shown to be effective in ameliorating the TNFα increase and cognitive impairment resulting from a mild traumatic brain injury in mice [36]. In the current study, 3,6′-DT and thalidomide were equally effective, at a high dose, in preventing CNS TNFα increases resulting from LPS-induced systemic inflammation in WT mice. However, only 3,6′-DT was effective at lowering TNFα in the CNS of 3 × Tg mice and improving cognitive function. The lack of efficacy by thalidomide in the 3 × Tg model may be due to a higher IC_50_ value than 3,6′-DT or a difference in brain penetrance. 3,6′-DT has an assessed brain to plasma ratio of 1.34 [36], which is in accord with its partition coefficient (cLogD) value of −0.56 [54,55], a measure of its balanced aqueous solubility and lipophilicity. Thalidomide has a moderate degree of lipophilicity with a cLogD value of −0.83 [56]. Further, thalidomide has demonstrated poor ability *in vitro* to reduce LPS-stimulated TNFα while significantly increasing toxic nitrite levels [36].

Within the CNS, the main executors of innate immunity are perivascular macrophages and parenchymal microglia [57]. Resting microglia are highly ramified with branched processes and have critical physiologic roles, including determination of neuronal fate, migration, axonal growth and synaptic remodeling [58,59]. In response to pathological conditions, microglia transform into a reactive state [58,60,61], losing their ramified morphology and switching from a neurotrophic phenotype to a persistent, reactive phenotype expressing toxic and reparative functions [59]. Inflammation clearly occurs in pathologically vulnerable regions of the AD brain [62,63], and both animal models and clinical studies strongly suggest that inflammation significantly contributes to AD pathogenesis [39]. 3 × Tg mice showed a large increase in Iba-1 positive microglia at 6.5 months of age compared with Non-Tg mice and the majority of these microglia had an activated morphology. Although both thalidomide and 3,6′-DT reduced the total numbers of Iba-1 positive microglia, only 3,6′-DT improved the ratio of resting to activated microglia and created a microglial morphological profile that was nearly identical to that of the Non-Tg brain. 3 × Tg mice treated with thalidomide had predominately activated Iba-1 positive microglia.

Although considerable focus has been given to the CNS-endogenous innate immune system, the CNS-endogenous and peripheral immune systems do not function in isolation from each other and there is dynamic interplay. In the healthy brain, peripheral immune cells are indispensable for homeostasis whereas specific cell types play different roles in neuroprotection and neurodestruction [64]. This activity must be tightly regulated or the immune system will become hyperactivated, leading to dysregulation and pathological sequelae. One of the many known functions of TNFα is to stimulate the recruitment of innate effectors such as neutrophils/granulocytes and monocytes and to activate these cells in a paracrine manner. After being activated by injury or disease, innate immune system functions focus on the clearance of pathogens and debris, tissue repair and in orchestrating adaptive immune responses [58,65,66].

While the inflammation within the AD brain has been assumed to be a locally-mediated inflammatory response by microglia to Aβ [39], systemic immune responses may also play a role. Prior to entering the CNS, leukocytes encounter formidable barriers to access including the endothelial blood–brain barrier, the epithelial blood-CSF barrier and the tanycyte barrier surrounding the circumventricular organs [67]. The mechanisms by which leukocytes infiltrate the healthy brain during adulthood and aging are not clear [68], but there is strong evidence that a compromised blood–brain barrier in brain diseases, such as stroke or brain trauma [69], and AD [70-75] allows leakage of leukocytes into the brain. TNFα can cause vascular endothelial damage and an increase in vascular endothelial permeability [76]. In regards to macrophage infiltration into the CNS, there are no specific immunohistochemical markers to distinguish blood-derived macrophages from brain endogenous microglial cells, making the distinction difficult. Recent human studies demonstrate that recruited monocytic cells express markers for microglia yet are morphologically and functionally separate from the resident microglia in the AD brain [71-75]. Contradicting the importance of leukocytes in AD pathology, histological examination of postmortem AD brains has not demonstrated abundant leukocytic infiltrates. Future studies and more sophisticated methodologies are required to determine if this is a disease stage phenomenon and what contributions infiltrating leukocytes may make to early stage AD and progression of the disease. In this regard, animal models of AD are valuable.

Recent studies in AD animal model pathology have elegantly examined the important role that brain-infiltrating monocytes play in Aβ clearance. Angelucci *et al*. crossed an AD mouse model with an animal deficient in CC-chemokine receptor 2 [77]. These bigenic mice have markedly diminished recruitment of brain resident microglia and/or peripheral macrophages to sites of beta-amyloid plaques and demonstrate heavier Aβ protein burden than AD model mice alone. While this study does not definitively establish the provenance of the amyloid clearing cells, it demonstrates the importance of brain innate immunity in restricting cerebral amyloidosis. An additional report has shown that depletion of CD11c + cells using a CD11c-diphtheria toxin transgenic mouse bone marrow chimera in an AD mouse model opposes the beneficial effect of T cell-directed immunotherapy, suggesting that peripheral innate immune cells are required for Aβ clearance [78]. These studies, by negatively impacting brain penetration and Aβ homing of these peripheral innate immune cells, lead to the deduction that such cells are critical for reducing amyloid accumulation. However, if these cells are to be targeted as a therapeutic modality, strategies for selectively increasing brain leukocyte infiltration and increasing Aβ clearance potential need to be developed. A recent study demonstrated that blood-borne monocytes can be encouraged to enter the brain and restrict Aβ plaques without producing a potentially damaging neuroinflammatory response [79].

Previous work [80] and the current study demonstrate that the 3 × Tg mouse has a robust increase in CNS leukocyte infiltrates early in the disease process, and that intracellular TNFα levels in this population are greatly increased relative to Non-Tg mice. Both thalidomide and 3,6′-DT reduced the total numbers of infiltrating peripheral leukocytes in the CNS of 3 × Tg mice as measured by flow cytometry but 3,6′-DT effected a striking reduction. The similar parallel finding of reduced Iba-1 microglia following thalidomide or 3,6′-DT treatment suggest that reducing the infiltrating leukocyte population contributed to the reduction in Iba-1 positive microglia. Additionally, only 3,6′-DT improved the resting to activated ratio of CNS microglia suggesting that the improved CNS penetrance and lower IC_50_ of 3,6′-DT compared with thalidomide is necessary for efficacy.

3,6′-DT did not, however, change the percentages of specific cell types within the total leukocytic population or alter TNFα levels in the total CD45_hi_ population. Rather, 3,6′-DT specifically reduced intracellular TNFα levels in the CD45_hi_/GR1^+^/Ly6G_hi_ (granulocytic) subpopulation. Due to a paucity of studies, it is unclear what the role of granulocytes is in the human AD brain, particularly in the early stages of the disease, and further studies are needed to determine if granulocytes migrate through the brain parenchyma or are involved in inflammatory signal transduction from the perivascular regions of the brain. Regardless, these data raise interesting questions about AD immunotherapy and suggests that, in addition to reducing the total number of infiltrating leukocytes, modulation of TNFα by small molecule TNFα inhibitors, in specific subsets of peripheral leukocytes, may be therapeutic.

Chronic neuroinflammation is an important component of AD pathogenesis and undoubtedly contributes to neuronal dysfunction, injury, loss and disease progression. A recent proteomic profiling study examined the CSF of young individuals who will go on to develop familial AD in comparison with age-matched controls not carrying a familial AD mutation [81]. The study noted increases in multiple complement cascade components as much as a decade prior to the onset of overt AD symptomology, indicating that neuroinflammation plays a very early role in the disease process. These and other data underscore the therapeutic potential of targeted anti-inflammatory pharmaceuticals both early and throughout the course of the disease. Unfortunately, our knowledge of CNS-related immune function is currently limited and the study of the interface between the peripheral and CNS-endogenous immune systems is in its infancy. Understanding the molecular manipulations required to produce beneficial changes in leukocyte and microglial activation profiles is necessary to beget more sophisticated immunomodulatory strategies for the treatment of AD.

## Abbreviations

3,6′-DT: 3,6′-dithiothalidomide; 3 × TgAD: Triple transgenic Alzheimer’s disease mice harboring PS1M146V, APPSwe and tauP301L; Aβ: Amyloid beta peptide; AD: Alzheimer’s disease; ANOVA: analysis of variance; APC: Allophycocyanin; APP: Amyloid precursor protein; cLog D: Partition coefficient; CM: Culture medium; CNS: Central nervous system; CSF: Cerebrospinal fluid; DMEM: Dulbecco’s modified Eagle medium; DMSO: Dimethyl sulfoxide; ELISA: Enzyme-linked immunosorbent assay; FACS: Fluorescence activated cell sorting; FBS: Fetal bovine serum; Iba-1: Ionized calcium-binding adapter molecule 1; IC50: Half maximal inhibitory concentration; IL: Interleukin; i.p: Intraperitoneal injection; LDH: Lactate dehydrogenase; LPS: Lipopolysaccharide; MCI: Mild cognitive impairment; Non-Tg: Non-transgenic; PBS: Phosphate-buffered saline; PCR: Polymerase chain reaction; PerCP: Peridinin-chlorophyll-protein-complex; PS1: Presenilin 1; RAM: Radial arm maze; Thal: Thalidomide; TNFα: Tumor necrosis factor-alpha; WT: Wild type.

## Competing interests

SPG and FPZ are employees of P2D, Inc. and declare a financial competing interest. All other authors declare no competing interests.

## Authors’ contributions

MEH-W participated in the design of the study, performed design-based stereology, isolated brain mononuclear cells, performed data and statistical analyses and drafted the manuscript. SPG participated in the design of the study, drug synthesis, data analysis and participated in drafting the manuscript. MKS and SPS conducted the flow cytometry work and subsequent data analysis. PE conducted all tissue extractions and ELISA and RT-PCR work. MFJ performed all immunohistochemical procedures, RAM studies and assisted with stereology. NK performed all cell culture and *in vitro* ELISA procedures. FPZ participated in the design of the study and in revising the manuscript. NHG is the primary inventor of the US patent covering 3,6′-DT (US patent 7,973,057 B2). DT and NHG made contributions to the concept of 3,6′-DT use in these studies, performed the chemical characterization and quality control of 3,6′-DT for cell culture studies, provided preliminary data on cellular and *in vivo* activity for appropriate dosing and participated in drafting the manuscript. All authors read and approved the final manuscript.
